# Zeolite-based nanoparticles drug delivery systems in modern pharmaceutical research and environmental remediation

**DOI:** 10.1016/j.heliyon.2024.e36417

**Published:** 2024-08-16

**Authors:** Tosha Pandya, Shruti Patel, Mangesh Kulkarni, Yash Raj Singh, Akruti Khodakiya, Sankha Bhattacharya, Bhupendra G. Prajapati

**Affiliations:** a L. J. Institute of Pharmacy, L J University, Ahmedabad, Sanand, Sarkhej-Gandhinagar Highway, 382 210, Gujarat, India; b Parul Institute of Pharmacy, Parul University, Lambda, Vadodara, 391760, India; c C.U. Shah College of Pharmacy and Research, C.U. Shah University, Surendranagar-Ahmedabad State Highway, 363030, Gujarat, India; d Department of Pharmaceutics, School of Pharmacy & Technology Management, SVKM'S NMIMS Deemed-to-be University, Shirpur, Maharashtra, 425405, India; e Shree S.K. Patel College of Pharmaceutical Education & Research, Ganpat University, Gujarat, India; f Gandhinagar Institute of Pharmacy, Gandhinagar University, Khatraj-Kalol Road, Moti Bhoyan, Kalol, Gandhinagar, 382721, Gujarat, India; g Faculty of Pharmacy, Silpakorn University, Nakhon Pathom, 73000, Thailand

**Keywords:** Zeolite based nanoparticles, Therapeutic potential, Targeted drug delivery, Drug stability, Safety considerations, Clinical trials

## Abstract

This review explores the potential of zeolite-based nanoparticles in modern pharmaceutical research, focusing on their role in advanced drug delivery systems. Zeolites, integrated into polymeric materials, offer precise drug delivery capabilities due to their unique structural features, biocompatibility, and controllable properties. Additionally, zeolites demonstrate environmental remediation potential through ion exchange processes. Synthetic zeolites, with modified release mechanisms, possess distinctive optical and electronic properties, expanding their applications in various fields. The study details zeolites' significance across industrial and scientific domains, outlining synthesis methods and size control techniques. The review emphasizes successful encapsulation and functionalization strategies for drug delivery, highlighting their role in enhancing drug stability and enabling targeted delivery. Advanced characterization techniques contribute to a comprehensive understanding of zeolite-based drug delivery systems. Addressing potential carcinogenicity, the review discusses environmental impact and risk assessment, stressing the importance of safety considerations in nanoparticle research. In biomedical applications, zeolites play vital roles in antidiarrheal, antitumor, antibacterial, and MRI contrast agents. Clinical trials featuring zeolite-based interventions underscore zeolite's potential in addressing diverse medical challenges. In conclusion, zeolite-based nanoparticles emerge as promising tools for targeted drug delivery, showcasing diverse applications and therapeutic potentials. Despite challenges, their unique advantages position zeolites at the forefront of innovative drug delivery systems.

## Highlights

• Zeolites in polymeric materials offer precise drug delivery due to their unique structure and properties.
• Zeolites aid in removing contaminants from wastewater through ion exchange processes.
• Synthetic zeolites with modified release mechanisms have distinct optical and electronic properties.
• Zeolites play a significant role in various industries with enhanced versatility.
• Zeolites are vital for antidiarrheal, antitumor, antibacterial agents, and MRI contrast agents in medical applications.

## Graphical abstract

**Figure:**
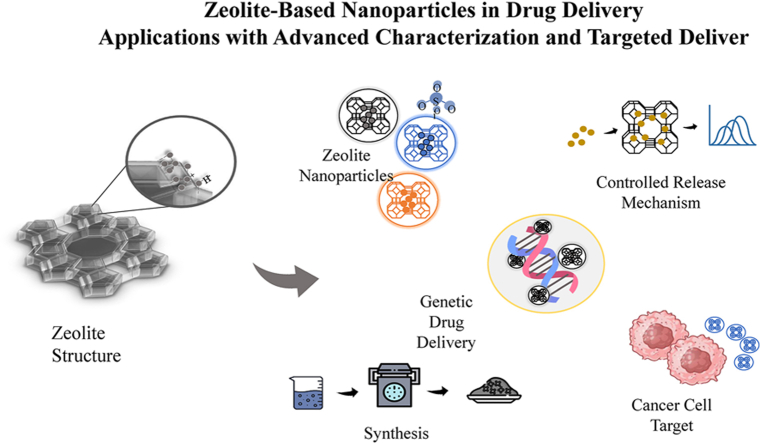

## 1 Introduction

Targeted and controlled drug delivery of therapeutics by designing various innovative functional drug delivery systems requires an hour. Liposomes, microspheres, dendrimers, solid lipid nanoparticles, polymeric nanoparticles etc., are among the multiple delivery systems being studied, but none of them make it to the clinic [1]. Micro- and mesoporous inorganic products, like zeolites, have drawn a lot of attention in research for specific and controlled drug delivery because of their distinct structural features, biocompatibility, large surface areas, and controllable physicochemical properties [2]. Zeolites are added to polymeric materials for the delivery of therapeutics in a variety of ways, including hosts, blends, composite materials, and gels. The uniform pore shape and ion exchange abilities of zeolites make them a focus of drug delivery systems, releasing drugs in a controlled manner. The highly crystalline structure of zeolites makes them suitable candidates in the formulation of hydrogels for therapeutic purposes [3]. They are selective in the absorption of wanted and unwanted organic or inorganic materials. Due to their morphology, they act as scavengers of toxic substances. In the biomedical field, zeolites are widely used due to their ability to be internalized in cells through endocytosis and their good biological durability, which can be used to deliver DNA to cells [[4], [5], [6]]. Apart from these advantages, zeolites are available in abundance and at affordable rates. Zeolites are characterized by chemical stability, biocompatibility, and modifiable structural characteristics. In addition, structures and their pore sizes vary widely, allowing them to exhibit high loading efficiency [7]. By interacting with the drug through ion exchange, zeolite enables the drug to be released rapidly by high surface absorption [8,9]. The superior ability of zeolite nanocomposites to efficiently load and release drugs makes them highly effective for biomedical applications over other porous materials [10].

The main challenge with zeolites is their tendency to cause cancer but this feature can be exploited in the treatment of cancer by utilizing its antiproliferative action.

### 1.1 Nanoparticulate drug delivery systems over conventional dosage forms

The various biological issues faced by drug molecules have paved the way for the development of nanosized drug delivery systems for these molecules. Small size and large surface area offer many advantages to nanoparticulate systems, like the ability to deliver substances with low solubility. Moreover, the drug molecules can be delivered to their target tissue or organ as the small size of Nano systems enables them to cross rigid cell borders [11]. Large-size molecules can also be efficiently entrapped in these systems, and drugs with different delivery rates can be simultaneously released from nanoparticles. Scientists are exploring various nanoparticulate drug delivery systems, e.g., liposomes, niosomes, lipid nanoparticles, microemulsions, dendrimers, polymeric nanoparticles, etc. Scientists have been interested in drug delivery systems (DDS) composed of organic materials for several years because of their biocompatibility and biodegradability [12]. Recent years have seen a significant increase in interest in inorganic materials for biomedical applications due to a number of intriguing qualities, such as their versatility in conjugation with various molecules, their tunable size and form, their biocompatibility, and their ease of functionalization [13]. It is crucial to look for a novel delivery system that won't have issues with the loading and release of drugs. One possible answer for this is a metal-organic framework (MOF), which offers the benefits of both organic and inorganic DDS [14]. Zeolite-like metal-organic frameworks (ZMOFs) are metal-organic frameworks (MOFs) that resemble conventional inorganic zeolites in terms of their topologies and, occasionally, characteristics. The remarkable qualities of this particular subset of MOFs result from their periodic pore systems and unusual cage-like cavities, along with their modular intra- and/or extra-framework components [15]. The main advantage of ZMOFs is the variety of pore sizes available which provides them with better drug loading capacity and also helps to control drug release [2]. The pore size and surface of zeolites can be modulated as per the drug delivery requirements.

### 1.2 Importance of zeolite-based nanoparticles

Zeolites are aluminosilicates that have an organized porous structure. They are divided into three main categories depending on the chemical composition of silica and aluminum: natural, synthetic, and zeolitic imidazolate framework [16]. The dense networks of ALO_4_ and SiO_4_ create mesoporous cavities and impart zeolites with their ion exchange characteristics [17]. The exterior surface area of zeolite crystals is increased for interaction with macromolecules as a result of their reduction to the nanoscale, which imparts the advantage to zeolites along with that of nanoparticles to combat severe diseases like cancer and aid in the controlled delivery of therapeutics [18]. A current active area of study is the management of zeolite nanoparticles' physicochemical characteristics to enhance the loading and delivery of medicinal medicines.

## 2 Search strategy and selection criteria

The focus of our paper was zeolites and zeolite-based nanoparticle drug delivery systems. For this review, online public databases with search engines that can handle specific keyword combinations were used to conduct a literature search on zeolites as a promising sector for nanoparticulate drug delivery systems. The selected web databases comprised PubMed, SCOPUS, Sci Direct, Google Scholar, Hindawi, clinicaltrials.gov, Google Patents, and Wiley online library. The following Boolean keywords were used: ((Zeolite) AND ((zeolite-based drug delivery) OR [19]) AND (((zeolite safety considerations) OR (nanoparticles))), AND ((zeolite based clinical trials) OR (zeolite framework)). The articles that made up this review were selected based on their pertinence to the topic. Furthermore, the selection included open-access articles that featured original research papers on in vivo and in-silico studies, randomized clinical trials, cohort studies, meta-analyses, and systematic reviews. Reference lists of published review articles were searched for additional relevant studies not identified in the database search. Experts in the field were contacted for publications not already identified. No restrictions were placed on the publication format to widen the base of literature to be reviewed on the topic; all journal articles including reviews, guidelines, and correspondence were included in the search. Publications from all countries were included but the language was restricted to English to avoid errors arising from incorrect translation.

## 3 Zeolite nanoparticles: types, synthesis and characterization

### 3.1 Types of zeolite-based nanoparticles

Zeolites exhibit a remarkable crystalline structure, characterized by their three-dimensional arrangement and porous Nature. These aluminosilicates possess an organized framework, consisting of tetrahedral units denoted as TO4, wherein an oxygen atom serves as a bridge between them; where T showcases Si or Al atom [20]. In the context of the zeolite framework, the substantial cavities within the structure play a crucial role in retaining water and facilitating the exchange of cations.

The chemical formula for zeolites is frequently referred to as:

M_a/n_ [Al_a_Si_b_O_2[21]_]. qH_2_O

**Fig. 1:**
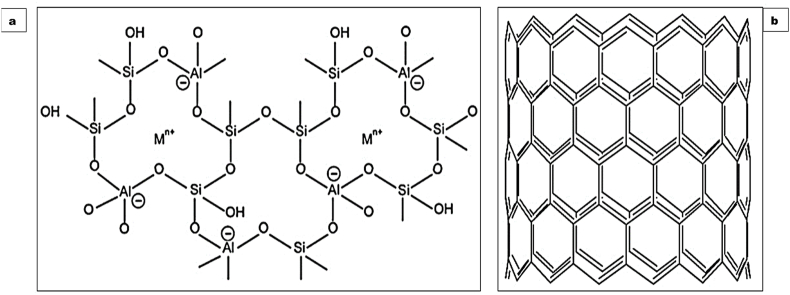
(a) Two-dimensional schematic representation of zeolite framework (b) Zeolite Skeletal Arrangement.

**Fig. 2:**
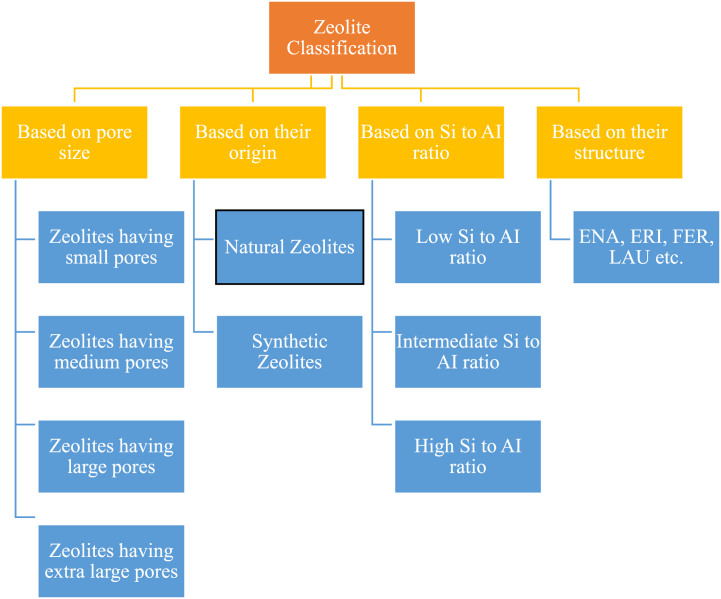
Classification of Zeolite based on different categories.

In this referred formula; M represents Sr, Ba, Ca, Mg and/or Li, K, Na, and cation charge is symbolized by n. The range of values for b/a spans from 1 to 6, while the range for q/a extends from 1 to 4. The two-dimensional schematic representation of zeolite framework is illustrated below in Fig. 1 [22]. Zeolites can be found in Nature, and they are also manufactured through chemical synthesis. The classification of Zeolites is illustrated in Fig. 2. The origin of natural zeolites can be traced back to volcanogenic sedimentary rocks. The presence of various zeolites in Nature is well-documented. Clinoptilolite, phillipsite, mordenite, chabazite, stilbite, analcime, and laumontite are a few of the more prevalent ones. These zeolites can be found abundantly in different geological formations. However, it is worth noting that certain zeolites, such as barrerite, offerite, and paulingite, are considered to be quite rare in occurrence [23]. The list of some naturally found zeolites along with their chemical formula is given below in Table 1.

**Table 1:** List of some naturally found zeolites along with their chemical formula.

Some Naturally occurring zeolite	Chemical Formula
Clinoptilolite	(Na_2_,K_2_,Ca)_3_Al_6_Si_30_O_72_·21H_2_O
Chabazite	(Na_2_,K_2_,Ca,Mg**)**Al_4_Si_8_O_24_·12H_2_O
Analcime	Na_16_Al_16_Si_32_O_96_·16H_2_O
Ferrierite	[26 Mg]_3_Al_6_Si_30_O_72_·20H_2_O
Laumontite	Ca_2_Al_8_S_16_O_48_·16H_2_O
Scolecite	Ca_4_Al_8_Si_12_O_40_·12H_2_O
Heulandite	Al_8_Si_28_Ca_4_O_68_·24H_2_O
Phillipsite	(Ca,Na_2_,K_2_)_3_Al_8_Si_10_O_32_·12H_2_O
Mordenite	(Ca, Na_2_, K_2_)Al_8_Si_40_O_96_·28H_2_O
Stilbite	Na_2_Ca_4_Al_10_Si_26_O_72_·30H_2_O

### 3.2 Fundamentals of zeolite-based nanoparticles

Drug development research revolves around zeolites due to their porous, surface-adjustable properties, which could be employed for regulated drug delivery. In recent years, nanoparticles derived from zeolites have drawn considerable attention as potential catalysts and drug delivery agents, and unlocking their potential in a number of sectors requires an understanding of their functionality [21]. These sections describe the fundamentals of zeolite-based nanoparticles.

Zeolites provide an ideal platform for the development of nanoparticles due to their unique structure. The porous Nature of zeolites provides an effective encapsulation and delivery system for drugs, while their crystalline composition ensures stability [24]. By encapsulating nanoparticles, zeolite prevents agglomeration and maintains its efficacy in varying conditions. Nanoparticles derived from zeolite exhibit impressive catalytic properties [25]. In recent years, nanoparticles derived from zeolites for use in drug delivery have gained popularity in the medical field due to their regulated pore size, which facilitates selective catalysis [26].Zeolites have been used in various petrochemical and chemical applications due to their regulated pore size. The porous Nature of zeolites makes them ideal for loading medicinal substances, and their controlled release allows for the delivery of medications to target areas where they are most effective. The tailored delivery approach enhances the efficiency of medicinal treatments while lowering the negative effects [2]. Although zeolite-based nanoparticles offer numerous advantages, they remain subject to several challenges, such as synthesis control and scalability, which must be addressed if they are to be commercialized widely.

1. Zeolites are known for their ion exchange capabilities. Using nanoparticles, this property can be utilized to remediate the environment and to purify water. Nanoparticles based on zeolite can selectively exchange ions with water, removing contaminants and pollutants.
2. Optical and Electronic Properties: The unique properties of zeolite-based nanoparticles extend into the realm of electronics and optics. Zeolite matrix confined spaces affect the electronic structure of encapsulated materials, resulting in tunable electronic and optical properties, which make them promising sources for applications in sensors and optoelectronic devices.

### 3.3 Characteristics of zeolites

Zeolites, crystalline aluminosilicates exhibit an excess of characteristics that render them crucially in various industrial and scientific applications. Previous studies were used to compile the following table, which offers the analysis of the crucial properties of zeolites. The various reported studies on different types of zeolites is presented in Table 2.

**Table 2:** Reported studies for type of zeolite applied.

Sr. No.	Zeolite type	Compounds	Carrier/Technique	Study	Reference
1.	Faujasite and Linde Type L	5-fluorouracil	Zeolite nanoparticles	Anticancer activity	[5]
2.	Beta and NaX- Faujasite zeolite	5-fluorouracil	silicalite-1 nanoparticles	Controlled release	[27]
3.	Faujasite and beta zeolite	Atenolol	MCM-41	Drug release and molecular dynamic simulation	[28]
4.	Zeolite Y	Sulfadiazine	Silver nanoparticles	Solid-state encapsulation	[29]
5	Clinoptilolite	Diclofenac sodium and indomethacin	Chitosan	Synthesis, characterization and drug release	[30]
6.	Surface modified clinoptilolite	Diclofenac and ibuprofen	Clinoptilolite	Drug release (ion exchange)	[31]
7.	Zinc-clinoptilolite	Doxorubicin	Graphene oxide nanocomposite	Synthesis and characterization	[32]
8.	Zeolite beta	Nifedipine	Zeolitic particles	Dissolution enhancement	[33]

### 3.4 Methods for zeolite nanoparticle synthesis

#### 3.4.1 Microwave and ultrasonic methods

**Fig. 3:**
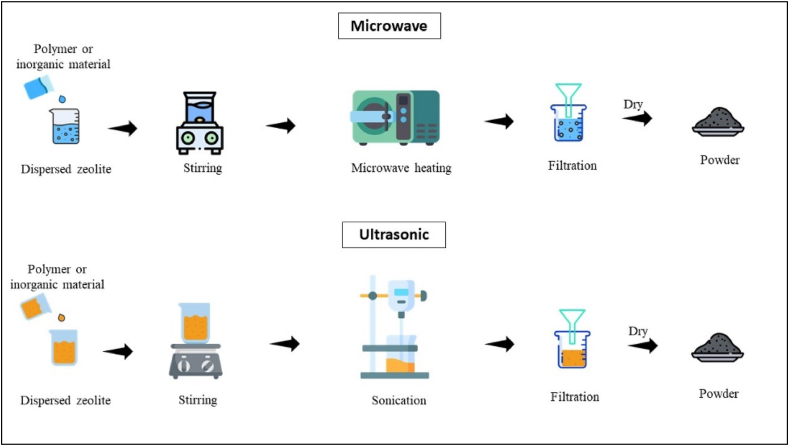
Preparation of Zeolite using Microwave and Ultrasonic Techniques.

Microwave and ultrasonic methods represent innovative approaches to the synthesis of zeolites, introducing unique and efficient means of achieving controlled and accelerated crystallization processes (Fig. 3). Microwave-assisted zeolite synthesis techniques harness the specific heating characteristics of microwaves, enabling rapid and uniform heating of the reaction mixture whereas the ultrasonic assisted synthesis of zeolites facilitates the dissolution of precursors and nucleation of zeolite crystals [34]. Several studies reported the synthesis of zeolites by coal fly ash through microwave and ultrasonic-assisted microwave irradiation [35] for samples with high solid-to-liquid ratio coal fly ash mass/NaOH solution volume with the process including sonication before [36,37]. The absence of conventional heating, along with continuous microwave irradiation, was observed to impede zeolite formation, as noted by the researchers. Additionally, the application of ultrasound energy after hydrothermal treatment enhanced the crystal growth of zeolite nuclei [38,39].

#### 3.4.2 Sol-gel and Co-precipitation methods

**Fig. 4:**
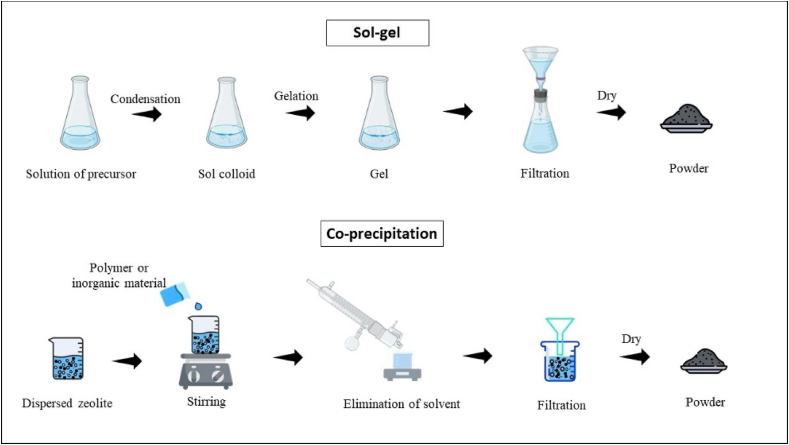
Preparation of Zeolite using Sol-Gel and Co-precipitation Techniques.

Sol-gel synthesis and co-precipitation stand out as distinctive methods in the scientific domain for crafting zeolites, each delineated by its unique processes and applications. Zeolite X particles were hydrothermally synthesized employing alumina sol by sol-gel process (Fig. 4). Alumina gel particles were aggregated near the micron-sized [40] zeolite X particles in an established microstructural study [41]. In sol-gel synthesis, the progression initiates with the hydrolysis and condensation of metal alkoxides as silica or alumina precursors, guiding a solution [42] through a transformation process into a gel that solidifies to produce the appropriate substance with the ability to simultaneously produce two or more types of nanoparticles [[43], [44], [45], [46]]. Co-precipitation synthesis orchestrates the simultaneous generation of multiple substances from a solution. In zeolite synthesis, co-precipitation unfolds through the amalgamation of solutions containing silicon and aluminum sources [47], where the pH of the solution serves as a pivotal factor initiating the precipitation process [48]. The precipitate undergoes aging, filtration, and drying before being subjected to calcination, ultimately conferring the zeolite structure. The microscopic study observed that the simplicity and cost-effectiveness methods of co-precipitation synthesis might involve slightly less control over the composition and crystallinity of the final product when compared to sol-gel techniques [49].

#### 3.4.3 Hydrothermal and solvothermal methods

**Fig. 5:**
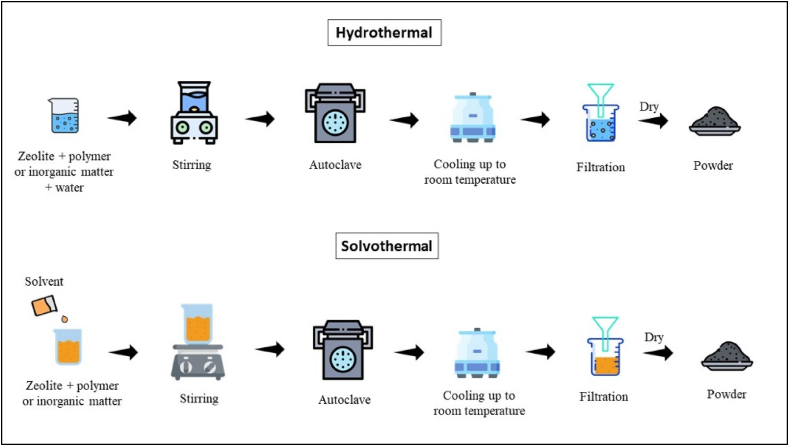
Preparation of Zeolite using Hydrothermal and Solvothermal Techniques.

The hydrothermal method involves the crystallization of aluminosilicate gels containing silica solution and aluminate mixture in the presence of organic bases and alkali hydroxides (Fig. 5) [50]. However, different factors highly affected the Nature and synthetic type of zeolite [51], such as alumina and silica composition, Nature of precursors and their pretreatment [52], temperature [53], pH, and reaction time of the reaction mixture [54]. The alumina and silica-based study of zinc-exchanged zeolite A synthesis by incorporating hydrothermal method for the formation of high crystallized zeolites [55]. Several studies have been reported and observed that the production of different types of zeolites was highly influenced by the final crystal structure and Si/Al ratio [56,57]. Solubilization techniques have been employed to surface passivate microporous zeolites in an effort to reduce the usual diffusional constraints. It has been reported that the addition of organic solvents makes it easier to control crystal growth [58]. By replacing the water-based crystallization medium with single-component or mixed organic solvents (like formamide, toluene, or toluene/butanol), which prevents aggregation and results in smaller (20–50 nm) and more homogeneous particle sizes, the dispersion of silanized zeolite seeds in the organic phase is enhanced [59,60].

### 3.5 Morphology and size control of zeolite nanoparticles

The importance of zeolite morphology in shaping catalytic activity, selectivity, and stability is undeniable, still establishing the precise quantitative descriptors for such morphology effects remains challenging. Structural characterizations were conducted to provide insights into the zeolites' morphologies, structures, and chemical properties, contributing to the ongoing effort to better define the intricate relationship between zeolite structure and performance [61]. Several organic template molecules have been employed in the synthesis process for altering the structure pore size and topology of zeolites by influencing parameters such as temperature, alkalinity, pH, stirring rate and reactant concentration [62,63]. Ephemeral exposure of zeolite crystals to hydrothermal and microwave radiation was reported to a reduction in crystal size with narrow particle size distribution [64,65]. Petushkov et al. [66] synthesized the Zeolite beta of Si/Al ratio of approximately 18 through a one-step hydrothermal treatment, allowing for the tailored development of isolated nanocrystals or mesoporous aggregates. The size control achieved resulted in zeolite beta nanocrystals ranging from 20 to 150 nm in mesoporous aggregates or diameter between 120 and 140 nm. Despite the varied morphologies, the crystallinity of the synthesized samples closely resembled of micron size zeolite beta which is commercially available. These samples exhibited remarkable surface areas exceeding 600 m^2^/g and mesopore volumes ranging from 0.4 to 0.5 cc/g. Similar study was conducted by Shi et al. [67] on ZSM-5 focused on regulating its morphology linked to diffusion properties. Thirty-five different additives, spanning diverse functional groups and polarities, were systematically screened. This approach successfully produced ZSM-5 with distinct morphologies, including sheet-like, plate-like, and noodle-like spherical structures, each exhibiting various sizes. Notably, the sheet like morphology demonstrated C4 olefin has cracking reaction as superior catalytic performance, as evidenced by the data of catalytic activity.

A novel gel preparation approach for all BEA-type silica crystals were involved in a two-stage process, with the I stage ensuring the formation of stable nuclei of BEA-type at a neutral pH, and the second stage controlling the number of viable nuclei through pH adjustment and additional aging in basic media, resulting a range of crystal sizes, from 2 to 15 μm, each with a distribution of narrow particle size was identified. Morphological variations, from well-developed shortened dipyramids to plate-like crystals, were achieved using the innovative synthesis method under basic conditions, providing extremely hydrophobic BEA-type crystals [68].

Hydrothermal synthesis of ZSM-5 zeolite was investigated with a specific focus on understanding the intricate relationship between zeolite morphology, Si/Al ratio, and catalytic behavior. The kinetic analysis of crystallization at Si/Al ratios of 20 and 100 revealed a notable transition in the synthesis process. As the Si/Al ratio rises, the sluggish dissolution-recrystallization process gives way to a quick solid-state change that elucidates the sensitivity of zeolite morphology to variations in the synthesis conditions and the consequential shift from clusters of slight agglomerates to larger circular particles underscores the profound impact of Si/Al ratio on the final zeolite morphology was observed [69]. A related study synthesized H-ZSM-5 series zeolites with a consistent sheet-like morphology but varying c-axis lengths [70]. The investigation revealed that the stability and catalytic activity were notably enhanced in samples with a longer c-axis. A combination of time-resolved in molecular dynamics simulations and in-situ FTIR spectroscopy and was employed to unravel the underlying mechanisms. The results demonstrated that the observed differences in anisotropy attributed by catalytic performance of the intracrystalline dispersive tendency of olefins in diverse channels within the zeolite structure.

### 3.6 Encapsulation and functionalization strategies

Since zeolites offer several noticeable characteristics, they are considered promising carriers for drug delivery. In a study, 5-fluorouracil, a drug used for cancer, was loaded into 2 different zeolite structures, Linde Type L and faujasite, to produce diverse drug delivery formulations [5]. In vitro assessments involving melanoma cell lines, breast cancer and colorectal carcinoma as well as in vivo studies utilizing the model based on chick embryo chorioallantoic membrane studies, were involved in the evaluation of the prepared formulation. Remarkably, both assays demonstrated optimal results for Hs578T breast cancer cells, with a notably enhanced efficacy observed for 5-fluorouracil encapsulated in zeolite Linde Type L. The study suggested that the caveolin-mediated process played a pivotal role in the internalization of zeolite nanoparticles. Another potentiation study of 5-fluorouracil employed the zeolites Faujasite and Linde type L with the conventional anticancer drug, hosted with different particle sizes, and successfully loaded 5-fluorouracil through characterization techniques. In vitro, drug release studies revealed rapid release kinetics, and the drug delivery systems significantly potentiated the effect of 5-fluorouracil on human colorectal carcinoma cell lines, HCT-15 and RKO. The zeolites-incorporated drug delivery system demonstrated no toxicity to cancer cells and facilitated zeolite-cell internalization, providing an enhanced multifaceted approach to the efficacy of traditional chemotherapy [71].

Moving to the second study, Souza et al. [72]explored the Faujasite zeolite channels with isoniazid adsorption, emphasizing the influence of pH on the adsorption process. Molecular modeling calculations provided insights into the drug-zeolite interaction, elucidating the geometrical disposition of drug molecules, saturation levels, and protonation states. The characterized hybrid material at pH 3, demonstrated its effectiveness as an isoniazid carrier, and the drug release study highlighted the stability of the zeolite carrier and potential application in antituberculosis treatment formulations.

The study represented a pioneering ideal perception for the utilization of metal-containing nanosized zeolites as carriers for hypercapnic/hyperoxic gases in the glioblastoma, addressing the challenge of hypoxia resistance in solid tumors in which the nanosized zeolite crystals exhibited a non-toxic profile in various living organisms, including non-human primates, mice, and rats [73].

### 3.7 Characterization techniques for zeolite-based nanoparticles

Zeolite nanoparticle characterization is continually advancing, with emerging analytical methods contributing significantly to its evolution over the past few years. These innovative techniques have enhanced our understanding and exploration of zeolite properties, paving the way for new insights and applications [74]. Zeolite-based nanoparticles exhibit complex porous structures with well-defined cavities and channels, and characterizing their structural features, including surface area, pore size distribution, and crystal size, is crucial for tailoring their performance in drug delivery, adsorption, and catalysis [75,76]. Detailed structural analysis of zeolite nanoparticles aids researchers in optimizing the synthesis methods and enhancing the nanoparticles' efficacy. The surface chemistry of zeolite-based nanoparticles plays a pivotal role in determining their reactivity and functionality. Fourier-transform infrared spectroscopy (FTIR) aid in identifying surface functional groups, supporting the design of materials with specific catalytic or adsorption properties [23]. X-ray diffraction (XRD) and nuclear magnetic resonance (NMR) unveil the crystal structure, enhancing the nanoparticle design with tailored pore sizes and configurations for optimal drug loading [77,78]. Techniques such as scanning electron microscopy and transmission electron microscopy provide insights into particle size distribution, growth rate, morphology, etc., which are considered crucial for tailoring nanoparticles for drug delivery applications, where considerations of particle size and shape profoundly impact cellular uptake and distribution [79,80]. Morphological structural changes were also reported when the zeolite and the Fe_3_O_4_ nanoparticles interacted in bonding [55]. Masaoki Iwasaki et al. [81]investigated the kinetics of NO_2_ desorption and states of NO_2_ adsorption on a Fe-loaded ZSM-5 zeolite employing temperature-programmed desorption and FTIR spectroscopy, revealed that some adsorption species (nitrate, NO^+^, NO_2_, nitrite) occurred; except for NO_2_, were considered to be formed via NO_2_ disproportionation and dimerization's reactions. The comprehensive understanding of their structural, morphological, and physicochemical characteristics is highly imperative for designing the effective drug delivery systems with enhanced encapsulation efficiency, controlled release, and improved biocompatibility.

## 4 Applications of zeolite-based nanoparticles in drug delivery and biomedical applications

### 4.1 Controlled release mechanisms

Biotechnology and biomedical science have developed quickly in the formulation of novel drug delivery, especially in genetic drug delivery [82]. Much research has proven that the liposomes, polymerases, and certain micro and mesoporous nanoparticles made up of inorganic material, i.e., zeolites, have increased the attention towards the development of control drug delivery systems [2]. Various types of zeolites, such as FAU, MFI, BEA, and CLI, have been employed effectively as drug carriers. Drug loading and encapsulation in various types of zeolite structures is affected by properties like pore size, surface area, Si/AL ratio, surface area, and hydrophobicity difference between drug and carrier material. Thermodynamic investigation revealed endothermic origin for the control encapsulation method, proving that the release profile from zeolite nanoparticles is within the dietary recommendation of humans [83]. Natural zeolites have the ability to deliver the nutrition or drug to the target site for a prolonged period of time due to its long-term stability and no degradability. Chabazite, a natural zeolite, is a tectosilicate mineral closely related to geminate. Carla Serri has modified chabazite using methylpyridinium chloride (CP), a cationic surfactant, better to understand the substance's release characteristics [42]. The findings show that the primary factors controlling drug delivery were film and particle diffusion coefficient; on the other hand, film thickness and distribution coefficient are significant factors in the film diffusion, and the effective diffusivity of the exchanging ions is significant in the particle diffusion. Additionally, clinoptilolite was used in an oral DDS as a porous platform for the release of different vitamins (A, D, and E). Such zeolites have buffering qualities that can extend the shelf life of vitamins and preserve them in acidic situations. Zeolites shield fat-soluble vitamins from the stomach's acidity, which explains why they exhibit significant bioactivity when present. To assess the release behavior, De Gennaro et al. treated the clinoptilolite with cetylpyridinium chloride and encapsulated with diclofenac sodium. Drug adhesion was governed by boundary layer diffusion and followed a pseudo-second-order reaction. Additionally, anionic exchange was used to modulate the drug release mechanism, and a quick final phase resulted in prolonged drug release.

Synthetic zeolite has proven advantageous over natural zeolite. This fact can be explained by a modified release mechanism or stimuli-sensitive drug release such as pH or the application of an electromagnetic field. Paradee and Sirivat have developed folic acid-encapsulated microporous structure of the zeolite Y alginate hydrogel [84]. The effect of an electric field on the drug's diffusion rate was examined, and it was discovered as the aluminium content increases the interaction between folic acid and zeolite increases, and it slower the drug release which is also called as “aluminium content effect”. Another term “Anode folic acid electrorepulsion,” is the electro-repulsive force that exists between a positively charged electrode and a positively charged FA in the presence of an electric field. As the Si/Al ratio of zeolite FAY rises, the electrical conductivity also increases. This results in a greater mobility of FA to diffuse from the matrix under an applied electric field. The percentage amount of crosslinkers also has effect on drug release as crosslinker concentration increases the smaller the size of pores and the release of the drug will be sustained.

Zeolite L was synthesized using polyvinyl alcohol (PVA)/pullulan cryogens to release enalapril maleate [85]. Zeolite's porous Nature allowed for an improvement in enalapril maleate loading efficiency. The addition of zeolite-L powder nanoparticles decreased the cryogens ability to absorb moisture and resulted in a new arrangement with respect to their morphology, which improved their releasing qualities. The Korsmeyer-Peppas release paradigm was followed by this system, which stipulates that diffusion processes regulate enalapril maleate release. In studies by Kocaaga et al., hydrogels made of Type A zeolites (Zn–Na ion-exchanged) and natural polysaccharide LM pectin were used for wound dressing, prolonging therapeutics [86]. This study demonstrated how oxygen transmission maintenance was improved by employing zeolites and inorganic porous materials. By using a membrane diffusion technique, zeolite A-containing hydrogels were synthesized, and they demonstrated the capacity to release the medication in a steady ratio of up to 86 % within 5 h. Zeolite A nanoparticles served as porous drug delivery systems, increased hydrogel stability via swelling, and maintained the rate of oxygen transport. Zeolite type A was also evaluated for antibacterial activity. Nitrous oxide riched zeolite A nanoparticle was evaluated for antimicrobial and wound healing properties against gram-positive and gram-negative bacteria. The rate at which NO was released was changed when the nano zeolite was added to a hydrophobic ointment base, which lowered the water diffusion characteristics. The addition of zeolite slowed the rate at which additional chemicals were adsorbed or distributed via its porous spaces, so speeding up the healing process in the treated area. It also controlled the discharge of stabilized ointment into the wound.

### 4.2 Theranostic applications of zeolite nanoparticles in cancer

Zeolites are intriguing theranostic platforms because they are porous inorganic materials that may be loaded with various medicines, photosensitizers, fluorescent dyes, radioactive tracers, gas bubbles, and enzymes [87]. Zeolites are widely recognized for their ability to trap different medications and imaging materials, as was previously mentioned in an application [88]. Furthermore, a number of researchers have demonstrated in recent years that zeolite may be successfully used for therapeutic purposes [89]. Recently, a hybrid combination of organic and inorganic compounds known as a metal-organic framework has emerged in the biomedical approach. A well-known MOFs, zeolite imidazole structure consisting of transition metal ions and imidazole, or imidazole derivatives, has determined application as cancer theranostic [90]. The structure of ZIFs has slight difference from the original structure of the natural zeolite, where role of oxygen has been replaced with imidazolate anions. As a result, ZIFs have the desired characteristics of both MOFs and zeolites at the same time. These characteristics include [19] high porosities and large surface areas because of the exposed edges of the organic linkers, which increase ZIFs' loading capacity [19]; ZIFs' ease of functionalization through the addition of active molecules or the modification of ligands; and [19] good chemical and thermal stability in organic solvents or alkaline water [91]. Furthermore, ZIFs have other intriguing features that are derived from their structure. In this manner, ZIFs can be stable in physiological circumstances due to the average strength of their conjunction bonds, but they degrade slowly in mildly acidic environments. Certain ZIFs, like ZIF-90, can decompose in response to subcellular adenosine triphosphate because of a more powerful conjunction between adenosine triphosphate and Zn2+. This allows ZIFs to achieve a controlled release of drugs and therapeutic molecules at the tumour site. Additionally, Zn2+ and imidazolate groups are biological system elements and have excellent biocompatibility. Because of the aforementioned qualities, ZIFs have emerged as exceptional contenders for a wide range of uses, including delivery of drugs, gas absorption, detecting, and catalysis. Particularly in the biomedical fields of tumour imaging, treatment, and biosensing [92]. For MRI diagnostics, for instance, the entrapment of iron oxide nanoparticles in porous zeolite nanocore has proven to be an effective platform as a contras agent. Zeolites have been used in cancer treatment in addition to the above-mentioned targeted treatment known as magnetic hypothermia, in which heat is produced when magnetic nanoparticles are subjected to an alternate magnetic field. They have concentrated on the zeolite's porous properties, which help to entrap magnetic nanoparticles and improve the T2MRI contrast enhancer even more. In this research, ferrous and ferric salt combined with NaY zeolite using a single pot technique creates the magnetic zeolite nanocomposite. The characterization of nanocomposite proves that it was successfully deposited in breast cancer cells, and at the highest concentration, it shows non-toxic Nature. In the end, the MZNC's capacity to function as an MRI imaging probe was verified and confirmed in vitro at a clinical field of 3T, producing a more excellent dark contrast than control cells. Zeolite nanoparticles are proven as effective carrier material for delivering cancer therapeutics such as doxorubicin paclitaxel to the targeted site. Fan Yang fabricated a ZSM-5 Zeolite/chitosan core-shell nano disk entrapped with doxorubicin to trat osteosarcoma [93]. The ZSM-5 zeolite and chitosan are fabricated into disc-like nanomaterials with a thickness of 100 nm and a diameter of 300 nm. The pore size of the mesoporous nano disc was 3.75 nm. The pH-responsive ZSM-5 Zeolite/chitosan nano disc has great drug loading up to 97.7 % and showed control drug releases fit to Kors Mayer papa's model. As demonstrated by pharmacokinetic studies, serological testing, and H&E staining assays, the DOX could be successful released from the ZSM-5/CS/DOX nano disks resulting from cellular endocytosis and induced apoptosis in cancer cells. In addition, the pH-responsive drug carriers resulted in effective tumor inhibition with minimal side effects, particularly cardiac toxicity.

In the second near-infrared window, Multi-model optical imaging shows an accurate, innovative platform for cancer theranostic. Ziliang Zheng et al. has established accurate PTT/catalytic synergistic treatment where zeolite-carbon-based nanozymes (HSC-2) were initially investigated as a tenable dual-modal NIR-II PA/NIR-II FL imaging nanotheranostics [94]. The electronic structure of zeolite nano-Beta containing 3D 12-ring pore system and a large surface area which can be changed from an indirect band gap to a direct band gap via doping carbon in the framework, resulting in excellent NIR-II FL emission characteristics, based on the adsorption capacity of ionic liquids. The efficacy of HSC-2 as a diagnostic and therapeutic tool is largely dependent on its cellular absorption. Cellular TEM analysis demonstrated that HSC-2 could be effectively endocytosed into cells during a 6-h incubation period with 4T1 cells and that the structure of HSC-2 was well preserved following cellular uptake.

### 4.3 Enhanced drug stability and bioavailability

Microporous minerals made from aluminosilicate, known as zeolites, are composed of an indefinitely expanding 3D structure of AlO4 and SiO4 tetrahedra connected to one another by the sharing of oxygens, creating a consistent network of channels. These channels and pores are used to entrap the drug molecules. Many researchers have proved that the entrapment of drugs in a zeolite framework improves the stability of drugs and bioavailability of drugs. Eleni Kontogiannidou et al. suggested that microporous faujasite zeolite could be successfully used for the entrapment of poorly water-soluble drugs [95]. Danazol was trapped in NaX-FAU using the incipient wetness technique for the aforementioned reason. By using UV spectroscopy and thermogravimetric analysis, the drug loading of danazol was assessed, and the results were in good agreement with the initial drug loading of 33.3 % w/w. XRD and thermometry were used to characterize the drug's crystallinity. The data indicates that the drug was highly crystalline and that the zeolite maintained its original structure during production. Isotherms used in texture analysis using the nitrogen sorption method maintain their distinctive form after the first wetness method, demonstrating the preservation of the zeolite microporosity. Under accelerated stability conditions, the formulation of zeolites demonstrates the loading and stability of encapsulated drugs for a 6-month duration. The dissolving profile of zeolitic particles encoded with danazol was evaluated in pH 1.2 simulated gastric fluid (SGF), fed state simulated intestinal fluid (FeSSIF), and fasted state simulated intestinal fluid (FaSSIF). The results indicated a progressive and increasing solubility of the medication in each of the three media. Ex vivo investigations utilizing the everted gut sac model demonstrate enhanced drug diffusion across rat intestinal membranes.

Rahimi et al. have synthesized a zeolite vitamin nano complex for vitamin encapsulation in zeolite [96]. They evaluated the stability and drug release of vitamins in zeolite powder. First, the powdered natural Iranian zeolite was dipped into a saturated solution containing vitamins A, D3, and E. After that, the powders were taken for 2 h, one, two, three, and four weeks at normal temperature in the laboratory. In the zeolite-free control sample, the extracted vitamin content steadily dropped with time, but in the zeolite-containing sample, it stayed unchanged. Because the zeolite had particles ranging in size from 710 to 850 μm, the amount of stable vitamins in the ambient environment after four weeks was greater than that of the control sample. The zeolite-containing sample released more than the control sample at the simulated gastric pH. Christina Karavasili et al. have performed a study on different zeolites named BEA, ZSM, and NaX [97]. To evaluate whether the zeolite nanoparticle can be effectively used as nanomaterials or not, they have synthesized indomethacin zeolite nanoparticle drug delivery. BEA and NaX showed high drug amorphization and high drug lading. Also, after stress testing, the drug properties, as well as drug loading, remained the same as the original. The drug release of indomethacin in stimulated gastric fluid and stimulated intestinal fluid at fasted and fed conditions was discovered to be dependent on alum inosilicate ratio of zeolite and degree of crystallinity of drug particles. The cytotoxicity assay was performed by MTT assay and flow cytometry assay suggested that zeolite nanoparticle effectively retained at targeted site and no toxic effect was observed.

Zahra Ahali Abadeh et al. evaluated curcumin's absorption property on zeolite type 5A [98]. They analyze that with an effective pore aperture of around 5 Å and the associated cation Ca2+ in the matrix, zeolite 5A facilitates a more stable and uniform drug delivery mechanism. According to UV–vis research, raising the drug content in the initial incubation solution increases the drug-loading effectiveness of curcumin in zeolite 5A. The presence of curcumin inside the zeolite pores and the increased interaction between the zeolite and curcumin at increasing loading concentrations were confirmed by DSC and XRD investigations, which supported this conclusion. Curcumin's presence in the zeolite structure was identified by FTIR analysis, which also determined the hydrogen bonding mechanism as the preferred interaction between curcumin and the zeolite carrier. Following curcumin loading, both SEM and XRD investigations demonstrated a stable zeolite structure that preserved its structural integrity. Li Linfeng et al. [99] carried out an investigation to find out how laying performance, tissue Zn accumulation, and the expression of Zn transporter genes in laying hens were affected by the zinc-bearing zeolite clinoptilolite (ZnCP), which is a substitute for zinc sulfate (ZnSO4). Hy-Line Brown laying hens were divided into three groups of six replicates, each containing fifteen hens. The groups were fed a baseline meal supplemented with ZnSO4 (80 mg Zn/kg diet, control), 0.23 % ZnCP (40.25 mg Zn/kg diet), and 0.46 % ZnCP (80.50 mg Zn/kg diet) for a period of eight weeks. Hens was fed a meal with 0.23 % ZnCP and showed comparable Zn levels in tested tissues when compared to the control group (P > 0.05). Zn buildup was increased in the pancreatic and liver (P < 0.05) with a greater ZnCP inclusion (0.46 %). Furthermore, blood iron [22] concentration rose with ZnCP inclusion (P < 0.05). The quantity of jejunal metallothionein-4 (MT-4) messenger RNA (mRNA) was increased by ZnCP supplementation (P < 0.05). Higher levels of ZnCP inclusion (0.46 %) raised the mRNA expression of zinc transporter-1 (ZnT-1) in the jejunum (P < 0.05) and MT-4 in the pancreas (P < 0.05). Hens given a 0.23 % ZnCP inclusion meal, had the greatest ZnT-2 mRNA abundance in their jejunum (P < 0.05). ZnCP was shown to have greater bioavailability than ZnSO4, as demonstrated by increased tissue Zn accumulation and Zn transporter gene expression.

### 4.4 Targeting ligands for specific cell interaction

To achieve effective treatment, many researchers have investigated targeted attached nanoparticles. The main drawbacks of conventional therapy is poor transport from barriers or membranes, stability, and insufficient drug release. Nowadays, dosage form stability and target ability are many researchers' hotspots. As a result, many innovative ligands and probes are available. It is worth noting a novel surface molecular imprinting technology. It is the process of attaching subtract to molecularly imprinted polymer. MIP with epitopes of proteins overexpressed in the tumor environment helps target tumor cells specifically.

Furthermore, it does not affect the drug release pattern of core nanoparticles, which attracts researchers to develop the targeted drug delivery. Recently, ya-ting qin has developed fluorescent zeolitic imidazolate framework (ZIF)-8 nanoparticles loaded with doxorubicin (FZIF-8/DOX) as the core and molecularly imprinted polymer (MIP) as the shell to synthesize tumor-sensitive biodegradable FZIF-8/DOX-MIP nanoparticles (FZIF-8/DOX-MIPs) [100]. The developed nanoparticle was effectively phagocyte by MCF7 cells and exert best inhibitory growth in tumor environment. Fre'de′ric Lerouge innovated Zeolite nanoparticles intended for laser-polarized 129Xe NMR studies [101]. To ensure that a peptide would be anchored for the purpose of targeting biological receptors and PEG chains would be attached for in vivo investigations, these nanoparticles were functionalized utilizing innovative synthesis techniques using semicarbazide COCHO chemistry. The outcomes showed that upon functionalization, the dissolved noble gas's accessibility to the micropores was preserved. To track the behaviour of zeolite nanoparticles in mice, scintigraphy has been studied with 111In attached to the particles. As initial evidence of concept for in vivo 129Xe MRI, these early 111In scintigraphy investigations demonstrated the location of the nanoparticles upon administration in mice and its biological distribution.

In the field of radionuclides, attaching to the bifunctional ligand is the main drawback. Nowadays, scientists are working on many nanotechnologies for the deployment of diagnostic therapeutic drugs. Nanoscale negatively charged zeolite particles can successfully bind with positively changed gamma emitter. The future of nanoparticle-based targeted treatment may be impacted by the development of new tools, particularly novel receptor-specific compounds, which are made possible by the reduction of zeolite particles from the micro-to nanometre size. Agata Piotrowskaolk [102] has worked on the sodium form of the type A zeolite synthesized with substance P, which can target the NK1 receptor of glioma cells. The high aluminum concentration in the structure of NaA nanozeolite explains why it has a much negative surface charge—more negative than other nanozeolites. Furthermore, the ζ potential stays negative following the conjugation of the peptide molecules. The NaA-silane-PEG-SP nanoparticles’ extremely low ζ potential value of −23.8 mV shows that the particles are unattached to one another and do not tend to aggregate. The 223RaA–silane–PEG–SP synthesized system was effectively attached to the NK1 receptor on glioma cells and exhibited a high cytotoxic effect. Radiometric analysis proves that there was no leakage of ^223^RaA from the Nano zeolite structure.

## 5 Toxicity and safety considerations

### 5.1 Biocompatibility and toxicity studies

Due to their high drug loading and inherent biodegradability, ZIFs have generated a lot of attention as pH-sensitive drug carriers. By altering polydopamine (PDA), ZIFs' biocompatibility is significantly increased, and the rate of degradability is also controlled [103]. One of the most exciting benefits of MOFs for biomedical applications, aside from the tailorability of the micro-nano hierarchical structure, is their biodegradability. Because metal ions and ligands are coordinated, MOFs have inherent biodegradability [104]. But with MOFs, biodegradability is a two-edged sword. On the plus side, MOFs may be entirely removed from the body through a tune deconstruction process without building up permanently in healthy organs. On the negative side, MOFs frequently degrade too quickly for in vivo applications, which frequently has substantial unfavorable side effects such as cell apoptosis, anomalies in significant organs, and even animal mortality [105]. A possible pH-sensitive transporter for anticancer medications is the ZIF subclass of MOFs. While it degrades under acidic circumstances, it is stable in physiological settings [106]. In order to accurately manage MOF disassembly and drug release profile, it is therefore much desired to govern MOFs biodegradability. This will allow for appropriate deterioration at the right pace and the removal of nanocarriers after drug delivery is complete.

Surface modification is crucial because it can control how materials degrade and enhance biocompatibility. The drug release is often aided by heat-facilitated diffusion in the described NIR-responsive drug-controlled release mechanism. ZIFs' degradation and biosafety were enhanced by the application of PDA, enabling its usage in in vivo investigations [103].

Host defence or antimicrobial peptides (AMPs) are crucial elements of numerous immune systems [107]. Broad-spectrum toxicity against bacteria, fungi, viruses, and malignancies has been demonstrated by several AMPs. Insect AMPs, in contrast to conventional chemotherapy medications, exhibit targeted cytotoxicity towards tumour cells while posing no overt harmful effects on healthy human cells. These properties work in concert to improve cancer treatment outcomes [108]. The broad-spectrum antimicrobial peptides known to suppress cancer cell line proliferation in vitro and in vivo are called Cecropins (CECs) [109]. ZIF-8 NPs, dubbed CEC@ZIF-8 NPs, were utilized in a study to load CECs with low bioavailability. It has been shown that encasing CECs in ZIF-8 NPs enhances the cytotoxicity and intracellular accumulation of cervical cancer cells [110].

Additionally, specific zeolites may have cytotoxic and carcinogenic properties. Erionite is a fibrous zeolite that resembles wool and is brittle. It shares characteristics with asbestos and can cause malignant mesothelioma and lung cancer [111]. It appears that specific structural characteristics, such as external surface area, pore size, surface charge, functional groups, and crystallinity, have an impact on the biosafety of these porous materials. The elemental makeup of zeolites is the cause of the toxicity problem in plants. The prevailing consensus is that aluminum is hazardous to plants because it causes oxidative stress, callose (1, 3-β-D-glucan) production, disruption of cytoplasmic Ca2+, and inhibition of root growth [112]. Less hazardous zeolites had a high Si/Al ratio. Dealumination is also thought to be a useful technique for lowering the amount of aluminium in synthetic zeolites [113]. One of the main causes of the production of reactive oxygen species, which increases toxicity, is the surface reactivity of these negatively charged zeolites. Fubini et al. investigated these zeolites' surface reactivity and how it affected the production of OH radicals [114].This issue can be overcome by coating the surfaces of zeolites, making them more biocompatible [115]. Because of the structure of the cells, choosing zeolites with a specific morphology—such as spherical or rod-shaped—will lessen their harmful effects; covering them with various polymers has no effect on porosity other than to stabilise their surface reactivity [116].

### 5.2 Toxicity and risk assessment of different zeolites used in drug delivery

Nanoparticles' reduced size may make them more harmful or alter the process by which they cause toxicity. Because manufactured nanoparticles and “incidental” nanoparticles differ in size, content, and surface characteristics, toxicological findings on the former cannot always be applied to the latter [117]. Because of the size- and shape-dependent features of nanomaterials that may impact toxicity, studies on their toxicity are complicated. It is possible to modify the mechanisms and degree of toxicity generated in cells by functionalizing silica-based nanoparticles [118]. Studies on the toxicity of crystalline silica related to quartz and zeolites that are significant to industry have been published in the literature [119,120]. Research on clinoptilolite, a naturally occurring zeolite, suggests that it is safe and harmless for humans and animals to use. However, other research has demonstrated that crystalline silica materials can be harmful by inhaling respirable-sized crystalline silica particles, which can induce silicosis [121].

Both erionite and mordenite have been demonstrated to induce fibrosis and mesothelioma in the lungs of mice; however, the effect is more noticeable in the case of erionite zeolite, which has thin fibers, as opposed to mordenite, which has granular and fibrous particles [122,123]. For zeolite toxicity research, one of the most often studied characteristics has been the impact of crystal shape. The crystal structure determines the morphology of zeolite crystals, which differ amongst them [124]. In certain instances, altering the synthetic conditions can regulate the shape of the zeolite crystals. The findings showed that the cytotoxicities of fibrous and nonfibrous dust with similar chemical compositions differ. When compared to nonfibrous mordenite, the fibrous zeolite erionite exhibited a noticeably higher rate of superoxide species formation [125]. Since toxicity varies depending on the particle size, porosity, shape, surface area, surface functionalization, and surface treatment, the literature presented here highlights the significance of proper material characterization.

## 6 Potential areas of growth and research

Zeolite nanoparticles (NPs) are known to have improved biocompatibility and adjusted biomolecular delivery capacity due to their unique structure and favorable physiochemical advantages over other mesoporous nanomaterials, including lower cytotoxicity, higher payload capacity, and improved intracellular targeting specificity and efficacy. Zeolites are used in many different biomedical applications, such as antidiarrheal, antitumor, antibacterial, and MRI contrast agents. They are also used in studies on bone formation, the development of Alzheimer's disease, and their hemodialysis, drug delivery, and dental applications [126]. Zeolites have a promising future in improving the quality of MRI pictures, as all nanosized porous materials have shown the ability to be used as MRI contrast agents [127]. Zeolites are high-spin metals that can attach to water molecules and produce proton spin relaxation durations that are noticeably faster. Zeolites have a regulatory role in the immune system and have a promising future as adjuvants for cancer treatment [128]. Furthermore, giving activated TMA-zeolite to diabetic and cancer patients reduces oxidative stress, which is linked to an improvement in overall health [129]. Medical pathologists are searching for novel approaches to combat the growth of multi-drug resistant bacteria, with reference to the evolutionary idea of growing inheritable bacterial resistance to traditionally prescribed organic antibacterial drugs. Among them, nanoscale solids are known to be efficient antibacterial agents because of their high surface area-to-volume ratio and low toxicity. The delivery of micronized zeolite (MZ) to a transgenic mouse model of Alzheimer's disease (AD) highlighted the antioxidant effects of MZ for age-related neurodegeneration, and the results showed no toxicity or negative consequences even over an extended length of time [130]. Unexpectedly, MZ treatment seemed to prevent amyloid genic processing of Aβ due to a considerable decrease in Aβ42 levels in MZ-treated transgenic mice.

In addition to being used to treat AD, zeolite nanoparticles have also been used to create AD biosensors and instrumentation [131]. When compared to micron-sized zeolites, nanoscale zeolites have an exterior surface area that is up to an order of magnitude bigger, indicating their potential for a wide range of uses in drug delivery systems. Moreover, targeting ligands may be used to further modify theranostic zeolite nanocarriers, which is very intriguing for targeted cancer therapy.

## 7 Clinical trials

Various studies on zeolite were compiled in Table 3.

**Table 3:** Clinical studies on Zeolite.

Sr.No.	Clinical study number	Zeolite type used	Summary
1.	**NCT03901989** [132] Treatment of Osteoporosis - TOP1 Clinical Study	zeolite and cellulose	This study investigates the effect of zeolite on bone mineral metabolism.
2.	**NCT00623675** [133] Analysis of Heavy Metals in the Urine of Twenty Well Individuals Using Mineralox	Dietary supplement: Mineralox C	Researchers want to know if using Mineralox, a zeolite (clinoptilolite) in conjunction with vitamin C, affects the levels of heavy metals in the urine. Twenty participants in the trial will get Mineralox from the study physician to evaluate if it aids in the body's elimination of heavy metals.
3.	**NCT01831492** [134] Effects of Zeolite + Dolomite on Performance and Acidosis	zeolite + dolomite device: cellulose	In this study, trained individuals' performance, exercise-induced acidity, oxidative stress, inflammation, and intestinal barrier dysfunction are examined in relation to dietary zeolite + dolomite.
4.	**NCT05178719** [135] Osteoporosis Treatment: TOP 2–5 Clinical Studies (Pear Control)	PMA-zeolite	With a minor modification to the research design, Protocol “TOP 2–5″ is an expansion of the TOP 1-study. Furthermore, since it is the primary prerequisite for approval by the FDA and EMA, prolonging the follow-up will offer a more dependable evaluation of the fracture risk, which is regarded as the most desirable outcome in the majority of clinical trials of osteoporosis treatment.
5.	**NCT04607018** [136] Impact of PMA-Zeolite on Blood Parameters and Mineral Metabolism (MMBP Study)	PMA-zeolite	A licenced medical device, the Panaceo Micro Activation [137] -zeolite releases alkaline ions (Mg2+, Ca2+, K+, Na+) while simultaneously absorbing specific hazardous chemicals (mostly heavy metals and ammonium ions) in the gastrointestinal system. In a recent study, the assessment of mineral metabolism was included as a side-effect, but no appreciable alterations were found. The major function of zeolite was determined to be to support and improve the intestinal wall barrier. To expand on the findings of earlier research, its potential for mineral release has to be further explored. The purpose of this human research was to measure certain blood parameters in order to expand this understanding.
6.	**NCT03817645** [138] Panaceo “MED” for IBS (Irritable Bowel Syndrome)	OTHER: Zeolite	Clinoptilolite is a kind of volcanic mineral that belongs to the zeolite group. There is a vast inner surface connected to the porous structure. Ions (such as Pb\^2+) can be exchanged or absorbed because of the anionic framework charge. As a class IIa medical device, the particular Panaceo PMA zeolite is authorised for the restoration of the intestinal lining. It satisfies applicable European standards and has a CE certification. The current investigation seeks to assess the following impacts on IBS patients: * Primary endpoint: impact on IBS symptoms; * Secondary endpoint: intestinal wall permeability and tight junction integrity as assessed by shifts in the concentration of zonulin in the stool. A prospective, double-blind, randomised controlled trial (RCT) is conducted in a monocentric, outpatient-controlled study with this goal in mind.
7.	**NCT04370535** [139] PMA-Zeolite-Clinoptilolite Effects in Crohn Disease	cellulose| PMA-zeolite	The purpose of this pilot trial is to assess the safety and effectiveness of PMA-zeolite in patients with uncontrolled Crohn's disease (Cd). A control group of healthy volunteers will get either PMA-zeolite or a placebo, and a test group of people with uncontrolled CD will receive either PMA-zeolite or a placebo. This will allow for a comparison of the effects of the PMA-zeolite. Furthermore, this pilot investigation ought to offer suggestions regarding the impact-size to calculate an effect for a potential follow-up human experiment.

## 8 Conclusion

The delivery of targeted and controlled drugs through innovative functional systems is essential in modern therapeutics. Due to their unique structural features, biocompatibility, large surface areas, and tuneable physicochemical properties, micropore and mesopore inorganic materials, including zeolites, have garnered considerable research interest. Through the combination of their small and large pores, metal-organic frameworks (MOFs) can offer both organic and inorganic drug delivery benefits. They are capable of captivating a good quantity of drug and releasing them over a prolonged period of time. Zeolites, with their porous Nature, are ideal for loading medicinal substances, allowing for targeted and efficient delivery. A variety of synthesis methods have been explored for zeolite nanoparticles (NPs), emphasizing their biomedical and theranostic applications. Zeolite-based nanoparticles exhibit complex porous structures with well-defined cavities and channels, and characterizing their structural features, including surface area, pore size distribution and crystal size, is crucial for tailoring their performance in drug delivery, adsorption, and catalysis. Despite their broad applications—ranging from antidiarrheal and antitumor treatments to antibacterial agents and MRI contrast agents—the primary challenge remains their toxicity. This can be mitigated by surface coating to improve biocompatibility. Zeolites are also utilized in bone formation studies, Alzheimer's disease research, drug delivery, and dental applications. This review presents various clinical trials involving zeolite-based nanoparticles. While they offer numerous advantages, challenges such as synthesis control and scalability must be addressed for widespread commercialization.

## Disclosure statement

The authors declare no conflict of interest.

## Funding

The authors received no funding for this work.

## Data availability statement

The authors confirm that the data supporting the findings of this study are available within the article references.

## CRediT authorship contribution statement

**Tosha Pandya:** Writing – review & editing, Writing – original draft, Software, Methodology, Investigation, Funding acquisition, Formal analysis, Data curation, Conceptualization. **Shruti Patel:** Writing – original draft, Visualization, Validation, Supervision, Software, Resources, Investigation, Formal analysis, Data curation. **Mangesh Kulkarni:** Visualization, Validation, Software, Project administration, Methodology, Funding acquisition, Data curation, Conceptualization. **Yash Raj Singh:** Supervision, Software, Project administration, Methodology, Investigation, Formal analysis, Conceptualization. **Akruti Khodakiya:** Supervision, Project administration, Methodology, Investigation, Funding acquisition, Data curation. **Sankha Bhattacharya:** Writing – review & editing, Writing – original draft, Supervision, Methodology, Data curation. **Bhupendra G. Prajapati:** Writing – review & editing, Writing – original draft, Visualization, Validation, Software, Resources, Investigation, Conceptualization.

## Declaration of competing interest

The authors declare that they have no known competing financial interests or personal relationships that could have appeared to influence the work reported in this paper.

## Abbreviations

AD: Alzheimer's Disease
AMPs: Antimicrobial Peptides
BEA: Beta Zeolite
BFLs: Bifunctional Ligands
CEC@ZIF-8 NPs: Cecropin-Loaded Zeolitic Imidazolate Framework-8 Nanoparticles
CLI: Clinoptilolite
CP: Methylpyridinium Chloride
CPM: Co-Precipitation Method
CRMs: Controlled Release Mechanisms
CTZ: Characterization Techniques for Zeolite
DDS: Drug Delivery System
EFS: Encapsulation and Functionalization Strategies
EOP: Electronic and Optical Properties
FAU: Faujasite Zeolite
FTIR: Fourier-Transform Infrared Spectroscopy
FZIF-8/DOX-MIPs: Fluorescent Zeolitic Imidazolate Framework-8/Doxorubicin Molecularly Imprinted Polymer Nanoparticles
HSC-2: Zeolite-Carbon-Based Nanozymes
HTM: Hydrothermal Method
IBS: Irritable Bowel Syndrome
IEC: Ion Exchange Capabilities
MACs: Mesoporous Cavities
MAS: Microwave-Assisted Synthesis
MFI: MFI Zeolite
MIPs: Molecularly Imprinted Polymers
MMBP: Mineral Metabolism and Blood Parameters
MOFs: Metal Organic Frameworks
MRI: Magnetic Resonance Imaging
MSC: Morphology and Size Control
MZ: Micronized Zeolite
MZNC: Magnetic Zeolite Nanocomposite
NATZs: Naturally Occurring Zeolites
NMR: Nuclear Magnetic Resonance
NPs: Nanoparticles
NPsDD: Nanoparticles Drug Delivery
PMA: Panaceo Micro Activation
PVA: Poly Vinyl Alcohol
RCT: Randomized Controlled Trial
ROS: Reactive Oxygen Species
SEM: Scanning Electron Microscopy
SGP: Sol-Gel Process
SMIT: Surface Molecular Imprinting Technology
SPNK1R: Substance P NK1 Receptor
STM: Solvothermal Method
SYNZs: Synthetic Zeolites
TEM: Transmission Electron Microscopy
TLSCIs: Targeting Ligands for Specific Cell Interactions
TMA: Tetramethylammonium
TNAPs: Targeted Attached Nanoparticles
TO4U: Tetrahedral Units (TO4)
TOP: Treatment of Osteoporosis
TPD: Temperature Programmed Desorption
TUNSF: Tunable Surface Features
UAS -: Ultrasonic Assisted Synthesis
XRD: X-Ray Diffraction
ZA: Zeolite A
ZBNPs: Zeolite-Based Nanoparticles
ZIFs: Zeolite Imidazolate Frameworks
ZSM-5: Zeolite Socony Mobil-5
